# A Decade-Long Retrospective Study of Factors Influencing Survival in Ruptured Abdominal Aortic Aneurysm

**DOI:** 10.3390/jcm13216431

**Published:** 2024-10-27

**Authors:** Günay Kalender, Thomas Weissmann, Ugur Dinç

**Affiliations:** 1 Department of Vascular and Endovascular Surgery, Vivantes Hospital Neukoelln, 12351 Berlin, Germany; guenay.kalender@vivantes.de; 2 Science & Research Department, Brandenburg Medical School Theodor Fontane, 16816 Neuruppin, Germany; 3 Department of Radiation Oncology, University Hospital of Erlangen, Friedrich-Alexander-University Erlangen-Nürnberg (FAU), 91054 Erlangen, Germany; thomas.weissmann@uk-erlangne.de

**Keywords:** abdominal aortic aneurysm, controlled hypotension, operative surgical procedures, aortic rupture

## Abstract

**Background:** Ruptured abdominal aortic aneurysm (rAAA) carries a high mortality risk, requiring rapid diagnosis and intervention. This study assesses various clinical factors influencing rAAA management outcomes in alignment with evolving guidelines from 2011 to 2024. **Methods:** A retrospective analysis of 62 rAAA patients treated at Vivantes Clinic, Berlin, from July 2014 to May 2024 was conducted. Data were obtained from medical records, focusing on vital parameters at admission and during treatment. Both numerical and categorical variables were analyzed to identify survival determinants. **Results:** The overall survival rate was 55%. Significant differences in systolic and diastolic blood pressures during surgery were noted between survivors and non-survivors, with lower pressures observed in non-survivors at critical surgical stages. Other vital signs showed no significant variations. Survival was significantly associated with gender, fluid therapy, and aneurysm location. **Conclusions:** Effective blood pressure management during surgery is crucial for improving survival in rAAA cases. This study emphasizes adherence to current clinical guidelines and highlights the need for ongoing research to fill existing knowledge gaps. Further investigations are essential to enhance patient care and outcomes in rAAA.

## 1. Introduction

Ruptured abdominal aortic aneurysm (rAAA) represents a critical medical emergency characterized by a notably high mortality rate, necessitating prompt and effective intervention. An abdominal aortic aneurysm (AAA) is defined by a localized dilation of the abdominal aorta, typically exceeding 3 cm in diameter. The risk of rupture is closely associated with the aneurysm’s size and growth rate, with aneurysms larger than 5.5 cm in diameter being particularly susceptible to rupture. The rupture of an AAA results in massive internal bleeding and shock, often leading to death if not promptly treated [1].

The European Society for Vascular Surgery (ESVS) updated its Clinical Practice Guidelines in 2024 to reflect the latest advancements and research in the management of AAAs [1]. These guidelines succeed previous versions from 2011 and 2019, providing a detailed, evidence-based approach to improving patient outcomes through standardized care practices [2,3]. Additionally, similar guidelines from the Society for Vascular Surgery (SVS) in North America contribute to the body of knowledge on AAA management [4].

These guidelines encompass extensive systematic reviews that critically evaluate between 500 and 1200 scholarly articles, synthesizing and categorizing recommendations based on the robustness of the underlying evidence and the strength of the recommendations provided. Consequently, they serve as the principal reference source for documenting and analyzing clinical experiences.

The prevalence of AAA is reported to be between less than 1% and 1.7%, with the rupture risk of AAA estimated to be less than 1% at one year for AAAs with a diameter of 50 mm, at four years for 40 mm AAAs, and at eight years for a 30 mm diameter of AAAs [1,5]. Analyzing data from various European countries and the USA, an annual hospital admission rate of 10 per 100,000 individuals has been reported for AAA [6]. This low incidence rate challenges the collection of large-scale empirical data, further complicating the establishment of best practices across healthcare provider groups.

Given the acute nature of rAAA presentations and the involvement of multidisciplinary teams, including paramedics, emergency responders, radiologists, anesthesiologists, and surgeons, standardizing protocols is challenging. The management of rAAA typically allows for limited time for thorough examination or comprehensive documentation, necessitating the continuous refinement and evolution of clinical guidelines.

As indicated in the summary table in Appendix A (see Table A1), particularly for cases involving rupture, the guidelines are subject to ongoing refinement and evolution. This dynamic progression may be attributed to the acute nature of the clinical presentations associated with such cases, which often allow only limited time for thorough examination or comprehensive documentation. Additionally, the management of ruptured abdominal aortic aneurysms (rAAAs) typically involves a multidisciplinary team including paramedics, emergency responders, radiologists, anesthesiologists, and surgeons, further complicating the standardization of protocols. Another factor influencing the continual update of these guidelines is the relatively low incidence rate of rAAA, which may affect the accumulation of large-scale empirical data.

Hence, the aim and scope of this study have been narrowed down to the most fundamental parameters while still encompassing the key factors relevant to the diagnosis, preoperative, intraoperative, and perioperative management of ruptured abdominal aortic aneurysm (rAAA). This approach ensures that the study remains focused on the most impactful aspects of patient care in this critical clinical scenario while maintaining the applicability and reproducibility of key outcomes across a wide range of settings.

This study aims to assess various clinical factors influencing rAAA management outcomes, analyzing data from patients treated over a decade at Vivantes Clinic, Berlin. By doing so, it seeks to contribute to the evolving understanding of rAAA management and inform future updates to clinical practice guidelines.

## 2. Materials and Methods

This retrospective study analyzed data from patients who presented with ruptured abdominal aortic aneurysm (rAAA) to the Department of Vascular and Endovascular Surgery at Vivantes Clinic Neukölln, Berlin, Germany. Data were extracted from hospital records, focusing on various physiological and clinical parameters at the time of admission and during the management of these patients. The study included both survivors and non-survivors of rAAA treatment.

Initially, all patients diagnosed with the code I71.3 between July 2014 and May 2024, according to the International Classification of Diseases (ICD), were included. There were initially 66 patients matching the criteria; however, 4 patients were excluded for the following reasons: one declined surgery after informed consent, one was deemed not suitable for anesthesia, access to one patient’s medical history was sealed, and one patient had additional conditions such as a duodeno-aortal fistula and aortitis, which could have biased the results due to their deviating nature.

The variables assessed included age; systolic and diastolic blood pressure (BP) at admission; pulse at admission; body temperature at admission; Glasgow Coma Scale score at admission; Quick Sequential Organ Failure Assessment score (qSOFA) at admission; blood oxygen saturation level (%O_2_) at admission; computed tomography (CT) scan evaluation of the size of the aneurysm (width, height); and initial, average, and final systolic and diastolic blood pressure readings during anesthesia. The categorical variables assessed included gender; anesthesia type; whether ultrasound guidance was used at admission; whether fluid therapy was administered at admission; history of previous surgery to manage rAAA; surgical approach (endovascular or open repair); usage of a balloon during surgery; renal artery proximity of the AAA (infrarenal or juxtarenal); and device type (bi-iliacal, mono-iliacal, or tube).

Descriptive statistics were computed for all variables. The distributions of each variable were compared between the survived and non-survived groups. Violin plots were used to visually inspect the distribution and spread of the numerical data, while bar plots were used for the categorical data.

To identify predictors of survival, we initially conducted univariate analyses using independent t-tests for continuous variables and chi-squared tests for categorical variables. Subsequently, a multivariate logistic regression analysis was attempted to control for potential confounding factors and to identify independent predictors of survival.

The statistical analysis and visualization of the data were accomplished using the Python programming language and relevant packages such as NumPy, Pandas, Matplotlib, SciPy, and Seaborn [5,7,8,9,10,11].

The following steps were taken in the analysis:

1. **Data Preprocessing**: The dataset was loaded using the pandas library, and columns were inspected to ensure completeness. Missing values were handled using median imputation, implemented via SimpleImputer from sklearn [9]. Categorical values such as “external”, “inpatient”, and “reanimation” were replaced with NaN to prepare the data for further processing.
2. **Categorical Data Transformation**: Categorical variables such as gender, anesthesia type, ultrasound guidance, fluid therapy, previous surgery, surgical approach, balloon usage, and prosthetic device type were converted to numerical format. This was performed using the get_dummies() function in pandas, which creates dummy variables for each categorical feature [9].
3. **Univariate statistical Tests**: Independent t-tests were applied to compare the means of continuous variables like systolic blood pressure and age between survivors and non-survivors. Chi-squared tests were employed to assess associations between categorical variables (e.g., gender, surgical approach) and survival outcomes. These tests were conducted using scipy.stats to generate the *p*-values necessary for determining significance [5,10].
4. **Multivariate statistical test**: This logistic regression model was implemented using the Logit function from the statsmodels package [12] to calculate odds ratios (ORs) and 95% confidence intervals (CIs). L2 regularization (Ridge regression) was applied using the LogisticRegression class from the linear_model module in the sklearn package with the liblinear solver and max_iter=1000 to ensure convergence [10].
5. **Visualization**: Violin plots were generated using seaborn to display the distribution of continuous variables by survival status, and bar plots were used to show the distribution of categorical variables [11]. Custom annotations were added to highlight statistical significance. The results of the t-tests and chi-squared tests were annotated on the respective plots to indicate the level of significance for each variable. The color palettes and point colors were customized using matplotlib to enhance visual clarity [7,8].

The analysis provided insights into the impact of various clinical and physiological factors on the survival outcomes of patients with rAAA, contributing to the evolving understanding of rAAA management.

## 3. Results

### 3.1. Population

**Figure 1:**
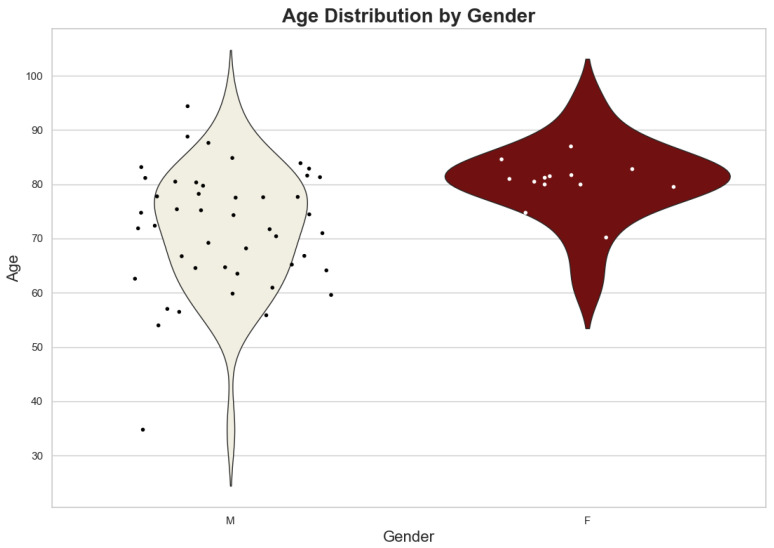
Age distribution by gender among the studied population. The violin plot shows the density of age distribution for male (M) and female (F) patients, with individual datapoints overlaid.

The initial population consisted of 66 patients, and after exclusion according to the criteria explained in Section 2, 62 eligible patients remained. The final population consisted of 46 males and 16 females, with an age range from 35 to 95 years. The mean age was 75 years. The age distribution by gender is visualized in Figure 1, illustrating that the majority of patients, both male and female, were aged between 65 and 85 years.

As shown in Figure 1, male patients exhibited a broader age distribution, while female patients were predominantly in the age range of 70 to 85 years. The overall pattern indicates that rAAAs were more common among older individuals in this cohort, with a slightly higher concentration of cases among males.

### 3.2. Mortality

The overall mortality rate in our dataset was 55% (N = 62). Of the 28 non-survivors, 10 died during surgery, 8 died on the same day as their surgery, 5 survived less than 30 days, and 5 survived between 30 and 60 days.

**Figure 2:**
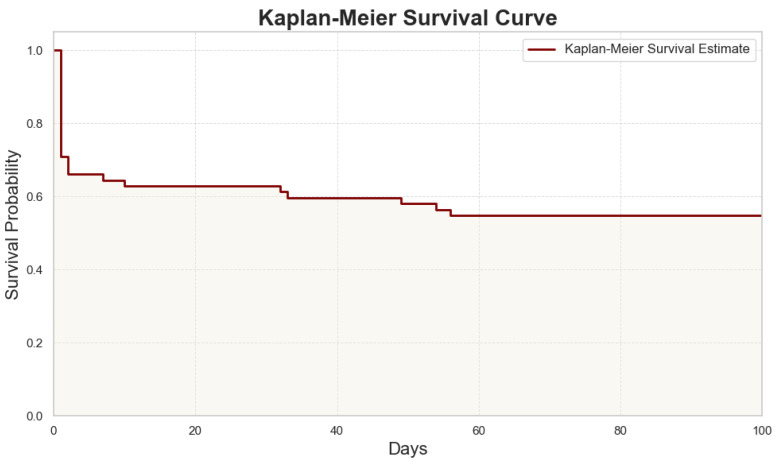
Kaplan–Meier survival curve showing the probability of survival over time for patients with ruptured abdominal aortic aneurysm. The shaded region represents the confidence interval for the survival estimate.

To illustrate survival differences over time, a Kaplan–Meier survival analysis was performed on the patient cohort. The Kaplan–Meier curve (Figure 2) shows the survival probability over time, with an upper limit of 100 days chosen to focus on the critical post-operative period.

The analysis reveals a sharp initial drop in survival probability during the first few days, which reflects the high-risk nature of this early period following ruptured abdominal aortic aneurysm (rAAA) repair. Patients who survived this initial critical phase displayed a relatively stable probability of survival thereafter. Notably, there were no patient deaths beyond 56 days during the monitored hospital observation, supporting the choice of 100 days as a clinically relevant upper limit for the plot.

### 3.3. Parametric Analysis

**Figure 3:**
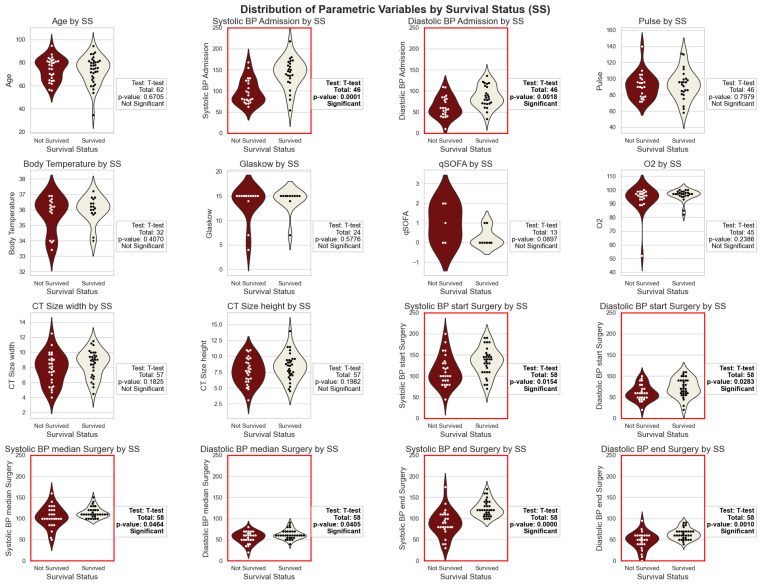
Violin plots showing the distribution of numerical variables by survival status for patients with ruptured abdominal aortic aneurysm (rAAA). The plots compare age, systolic and diastolic blood pressure at admission, pulse, body temperature, Glasgow Coma Scale score, qSOFA score, blood oxygen saturation level, aneurysm size (width and height), and blood pressure readings at various stages during surgery. Significant differences (*p* < 0.05) are indicated with a red outline, and the number of entries is listed above each subplot.

Key findings from the univariable analysis are summarized in the violin plots (Figure 3).

- **Systolic and Diastolic Blood Pressure (BP) at Admission and during Surgery:** Significant differences were observed in systolic and diastolic BP readings at various stages. Systolic and diastolic BP at admission were significantly lower in non-survivors (*p*-values: 0.0001 and 0.0018, respectively). During surgery, initial (systolic BP start: *p*-value: 0.0154, diastolic BP start: *p*-value: 0.0283), average (systolic BP median: *p*-value: 0.0464, diastolic BP median: *p*-value: 0.0405), and final (systolic BP end: *p*-value: 0.0000, diastolic BP end: *p*-value: 0.0010) BP readings were significantly lower in non-survivors.
- **Aneurysm Size (Width and Height):** Aneurysm size measured by CT imaging (width and height) showed no significant differences between survivors and non-survivors (width: *p*-value: 0.1825, height: *p*-value: 0.1982).
- **Other Parameters:** No significant differences were found for other parameters such as age (*p*-value: 0.6705), pulse (*p*-value: 0.7979), body temperature (*p*-value: 0.4070), Glasgow Coma Scale (*p*-value: 0.5776), qSOFA score (*p*-value: 0.0897), and blood oxygen saturation level (%O_2_: *p*-value: 0.2386) at admission.

The significant differences in blood pressure measurements indicate that lower blood pressure readings are associated with poorer outcomes in patients with rAAA, highlighting the importance of the close monitoring and management of blood pressure.

### 3.4. Categorical Analysis

**Figure 4:**
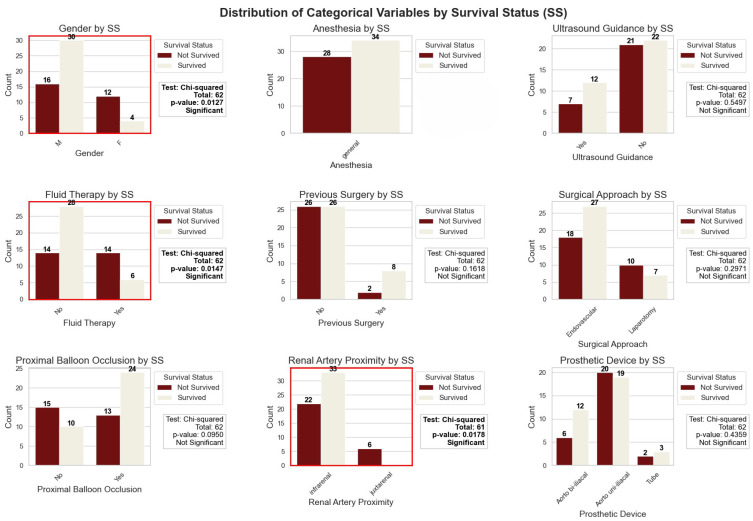
Bar plots showing the distribution of categorical variables by survival status in patients with ruptured abdominal aortic aneurysm. Variables include gender, type of anesthesia, use of ultrasound guidance, fluid therapy, history of previous surgery, surgical approach (laparotomy vs. endovascular), use of aortic balloon occlusion, location of the aneurysm (infrarenal vs. juxtarenal), and type of device used (Aorto bi-iliacal, Aorto uni-iliacal, Tube). Statistically significant differences (*p*-value < 0.05) are highlighted with red borders around the plots.

Bar plots showing the distribution of categorical variables by survival status are presented in Figure 4.

Key findings include:

- **Gender:** A significant difference was found between the survival status of male and female patients (*p*-value: 0.0127). More female patients did not survive compared to male patients.
- **Anesthesia:** Only general anesthesia was used in our cohort; hence, a comparison was not possible.
- **Ultrasound Guidance:** No significant difference was found in the use of ultrasound guidance at admission between survivors and non-survivors (*p*-value: 0.5497).
- **Fluid Therapy:** A significant difference was observed in the use of fluid therapy at admission (*p*-value: 0.0147). Non-survivors were more likely to have received fluid therapy compared to survivors.
- **Previous Surgery:** No significant difference was found in the history of previous surgery between survivors and non-survivors (*p*-value: 0.1618).
- **Surgical Approach:** No significant difference was observed between the types of surgical approaches (laparotomy vs. endovascular) used for treatment in relation to survival status (*p*-value: 0.2971).
- **Balloon Usage:** No significant difference was found in the use of aortic balloon occlusion between survivors and non-survivors (*p*-value: 0.0950).
- **Renal artery proximity:** A significant difference was noted in the proximity of the aneurysm with respect to the renal artery (infrarenal vs. juxtarenal) between survivors and non-survivors (*p*-value: 0.0178). Juxtarenal aneurysms were associated with higher mortality.
- **Prosthetic Device:** No significant difference was found in the type of device used (Aorto bi-iliacal, Aorto uni-iliacal, Tube) between survivors and non-survivors (*p*-value: 0.5740).

### 3.5. Multivariate Analysis

A multivariate logistic regression analysis was conducted using the Logit function from statsmodels [12] to identify independent predictors of survival in patients with rAAA. Variables that were significant in the univariate analysis, along with other clinically relevant factors, were included in the model.

However, the model was prematurely terminated before converging, and the results were not presented to avoid potentially misleading conclusions. The analysis encountered several challenges, including the high number of categorical and continuous variables, as well as significant collinearity between individual systolic and diastolic BP and among different BPs recorded at admission and during surgery. Furthermore, the unequal distribution of certain categorical variables, such as the absence of survivors among the three juxtarenal aneurysm cases, further complicated the model’s performance.

This analysis was particularly challenging from a technical standpoint, as models like these typically require a large number of datapoints, with each datapoint containing all relevant variables. In the case of an emergent and rare clinical condition like rAAA, both the total number of datapoints and the completeness of the data were limited. For instance, some patients were already hospitalized, meaning no admission recordings were available. Others, unfortunately, did not survive long enough for surgery, so while admission, demographic, and radiological data (e.g., aneurysm size) were available, the surgical data were inherently missing.

While an alternative approach could have been to trim problematic categories, this would have undermined the purpose of a multivariate analysis, which is designed to account for multiple interacting factors. Reducing the model to a limited set of variables would compromise its ability to provide meaningful insights into the complex factors influencing patient survival.

Given the emergent and rare nature of rAAA, single-center studies like this can lay a valuable foundation for future meta-analyses or multi-center studies, providing more statistically robust insights. With larger sample sizes and a more balanced distribution of variables, future studies will be able to draw stronger conclusions. Nevertheless, these early findings highlight the critical importance of vigilant hemodynamic management and strategic surgical planning in improving outcomes for patients with rAAA.

## 4. Discussion

Our findings underscore several critical factors associated with survival in patients with ruptured abdominal aortic aneurysm (rAAA), offering valuable insights into the evolving management strategies outlined in the ESVS 2024 guidelines [1]. The most general finding, which was the overall mortality rate in our dataset, at 55% (N = 62), was consistent with the reported literature rates. Specifically, previous studies have documented mortality rates ranging from 67 to 94% [13], 65 to 85% [14], and 48.5% [15].

### 4.1. Blood Pressure Management

The significant differences in systolic and diastolic blood pressures between survivors and non-survivors highlight the crucial role of blood pressure management. The guidelines consistently recommend permissive hypotension, although the level of evidence is graded as C, based on expert consensus and small, retrospective studies [1]. Prior studies have noted that hemodynamic conditions at admission are inconclusive regarding survival [16]. Conducting randomized controlled trials in rAAA settings is ethically and methodologically challenging, as noted in several reviews that highlight the lack of clinical or randomized studies on permissive hypotension [1]. Our findings suggest that median blood pressure during surgery converges around 120 mmHg for systolic pressure, implying that low blood pressure at admission may indicate deteriorating cardiovascular stability. Therefore, permissive hypotension might be indirectly supported, considering the extended operation times and close blood pressure monitoring by anesthesiologists.

### 4.2. Admission Vital Parameters

At first glance, it might be surprising to see that the pulse, O_2_ saturation, Glasgow Coma Scale, qSOFA score, and aneurysm width and height are not relevant to survival. However, this might indicate the binary implication of an aneurysm—if an aneurysm is ruptured, only blood pressure remains as a reliable parameter to monitor prognosis, while other parameters might be more relevant for monitoring an AAA for the risk of rupture.

### 4.3. Gender Differences

Gender differences play a significant role in the epidemiology and outcomes of abdominal aortic aneurysms (AAAs). While the prevalence of AAAs is consistently lower in women, studies show that when aneurysms do occur, they may carry a higher risk of rupture and worse outcomes compared to men. A systematic review of population-based studies between 2000 and 2015 reported that the pooled prevalence of AAA in women over 60 years was only 0.7%, up to fourfold lower than in men [17]. The higher threshold diameter of 30 mm used to define AAAs might be less appropriate for women due to their generally smaller aortic dimensions. This has led to suggestions for using lower thresholds or alternative criteria, such as body surface area-based definitions, to improve diagnostic accuracy in female populations [18].

Despite lower incidence rates, females with AAAs face worse prognoses. Studies indicate that women have a higher risk of aneurysm rupture and increased perioperative mortality following repair [19,20]. The reasons for these disparities may include differences in baseline aortic anatomy, hormonal influences on connective tissue integrity, and delayed diagnosis, as aneurysms in women are often detected at a more advanced stage. Moreover, smoking, the strongest risk factor for AAAs, carries an even higher relative risk in women compared to men [19,20,21].

In this study, we observed higher mortality rates among female patients with ruptured AAAs (rAAAs), consistent with findings from the previous literature. Future research should aim to better understand the underlying factors contributing to this disparity by incorporating more detailed hemodynamic monitoring, genetic profiling, and hormonal assessments. Such an approach could lead to more personalized treatment strategies, addressing the specific risks and treatment responses of male and female patients with AAAs [20,21,22,23,24].

### 4.4. Fluid Therapy

The significant impact of fluid therapy on survival outcomes emphasizes the importance of optimizing resuscitation strategies. This aligns with the evolution of the guidelines as, except for the very first guidelines in 2011, all the subsequent guidelines have dropped this recommendation [1,2,3,4]. Based on our clinical experience, we follow a strict protocol for the differential diagnosis of rAAAs, ensuring that the on-call vascular surgeon is promptly notified and emergency surgery is initiated if necessary. Consequently, in our practice, fluid therapy does not cause any delay in proceeding to surgery.

### 4.5. Renal Artery Proximity

The significant difference in survival rates based on aneurysm location (infrarenal vs. juxtarenal) suggests that anatomical considerations should play a crucial role in treatment planning, as highlighted in the latest guidelines [1]. Juxtarenal aneurysms were associated with higher mortality, indicating that these patients require more aggressive and perhaps innovative treatment approaches.

### 4.6. Other Factors: Ultrasound Guidance, Previous Surgery, Surgical Approach, Balloon Usage, Aneurysm Size and Prosthetic Device

Ultrasound guidance, previous surgeries, endovascular approaches versus laparotomy, balloon occlusion, and the choice of prosthetic device are all practices recommended and expected to improve survival rates in rAAA management [1]. In our study, statistical tests did not show significant differences for these variables. However, this should be interpreted with caution, as the absence of evidence does not necessarily imply evidence of absence. In each of these cases, there was an observed trend towards higher survival rates. The lack of statistical significance may be attributed to the relatively small cohort size. Therefore, these results should be regarded as inconclusive rather than negative, highlighting the need for further research with larger sample sizes to definitively determine their impact on survival.

### 4.7. Comparison with Selected Further Studies

In the present study, there was no difference in mortality between open and EVAR for rAAAs, which is not consistent with some larger reports. For instance, a review of the Nationwide Inpatient Sample dataset found higher all-cause 30-day readmission rates following EVAR compared to open repair [25]. Another review of the Vascular Quality Initiative found that open repair was associated with higher odds of hospital mortality compared to EVAR for ruptured AAAs [26]. Furthermore, our findings that survival was worse for women compared to men contrasts with other reports showing higher mortality rates in females after rupture repair [27,28]. These discrepancies highlight the need for further studies to validate our findings and explore the reasons for these differences.

### 4.8. Limitations

While this study provides valuable insights into the factors influencing immediate and short-term survival in ruptured abdominal aortic aneurysm (rAAA), it is important to note the limitation related to the absence of long-term outcome data. The lack of follow-up beyond the initial post-operative period restricts our ability to evaluate the durability of surgical repairs, the occurrence of late complications, and the overall quality of life of patients. Long-term data could offer further insights into patient recovery trajectories and the extended efficacy of different management strategies. Future studies should consider incorporating long-term follow-up to address these aspects and provide a more comprehensive understanding of rAAA outcomes.

Given that this study is a single-center experience, a brief discussion of the general surgical technique at our institution is warranted. Our protocol includes immediate transfer to the operating room for patients with confirmed rAAA, with a preference for endovascular repair whenever anatomically feasible. The choice of surgical approach and prosthetic device is guided by the patient’s clinical condition and anatomical considerations.

## 5. Conclusions

This study underscores the critical role of effective blood pressure management during surgery for patients with ruptured abdominal aortic aneurysm (rAAA), demonstrating that lower systolic and diastolic blood pressure readings are associated with poorer survival outcomes. These findings support the use of permissive hypotension as recommended by current guidelines, emphasizing the need for close hemodynamic monitoring and tailored resuscitation strategies in rAAA management.

Additionally, the results suggest that anatomical considerations, such as the location of the aneurysm, should be prioritized in surgical planning, with juxtarenal aneurysms requiring more aggressive interventions. The observed gender differences in outcomes further highlight the need for personalized approaches, potentially involving gender-specific treatment protocols to improve patient survival.

This study’s findings may influence clinical guidelines by advocating for refined protocols that incorporate detailed risk assessments based on blood pressure management, aneurysm anatomy, and patient demographics. Further research should focus on integrating these factors into standardized treatment algorithms, ultimately enhancing decision-making processes and optimizing outcomes in rAAA care. Continuous refinement of guidelines is essential, given the evolving nature of rAAA management and the complexities involved in treating such critical cases.

## Data Availability

Due to the limited number of patients and privacy concerns, the data is not publicly available.

## Abbreviations

The following abbreviations are used in this manuscript:

AAA	abdominal aortic aneurysm
ACS	abdominal compartment syndrome
BP	blood pressure
CT	computed tomography
ESVS	European Society for Vascular Surgery
EVAR	endovascular aneurysm repair
eVR	endovascular repair
ICD	International Classification of Diseases
%O_2_	blood oxygen saturation level
oeVT	open or endovascular treatment
OP	operative
P	page number
qSOFA	quick sequential organ failure assessment
R	recommendation number
rAAA	ruptured abdominal aortic aneurysm
SS	survival status
SVS	Society for Vascular Surgery

## Appendix A. Guideline Evolution Summary

The following table summarizes the evolution of recommendations regarding ruptured abdominal aortic aneurysms (rAAAs), as referenced in the main text. This table provides additional details that supplement the information in the body of the paper.

**Table A1:** Evolution of recommendations from major guidelines.

Latest Formulation * of the Recommendation	2024 ESVS	2018 ESVS	2018 SVS	2011 ESVS
The summary table above lists the evolution of the recommendations regarding rAAAs over time listed in Table A1. Patients with a suspected rAAA should undergo prompt imaging of the thoraco-abdominal aorta and of the access vessels with computed tomography angiography.	R70	R63	x	x
After endovascular repair (eVR) of abdominal aortic aneurysm rupture into the inferior vena cava, subsequent endovascular closure of the aortocaval fistula may be considered in the presence of an endoleak associated with increased cardiac output, heart failure, or pulmonary embolization.	R71	x	x	x
For patients with rAAAs, a policy of permissive hypotension is recommended.*	R72	R66	P41	P26
For patients undergoing an eVR of an rAAA, local anesthesia should be considered as the anesthetic modality of choice, whenever tolerated by the patient.	R73	R67	x	P28
Hemodynamically unstable patients with an rAAA undergoing open or eVR may be considered for aortic balloon occlusion under fluoroscopy guidance to obtain proximal control.	R74	R68	x	P28
Patients undergoing eVR for an rAAA may be considered for a bifurcated device, in preference to an aorto-uni-iliac device, whenever anatomically suitable.	R75	R69	x	x
Patients undergoing eVR for an rAAA in whom imaging was performed during permissive hypotension should be considered for a stent graft oversizing of up to 30%.	R76	x	x	x
In rAAA repair, intra-OP administration of systemic anticoagulation with heparin should be considered once the rupture bleeding has been controlled.	R77	x	x	x
Patients with an rAAA should be considered for post-OP deep vein thrombosis prophylaxis with low molecular weight or unfractionated heparin unless there are signs of ongoing bleeding or of a clinically significant coagulopathy.	R78	x	x	x
Selection of patients with rAAAs for palliation based entirely on scoring systems or solely on advanced age is not recommended.	R79	R70	x	x
For patients with an rAAA and suitable anatomy, eVR is recommended as the first-line treatment option.	R80	R74	P42	P28
After oeVT for an rAAA, post-OP monitoring of intra-abdominal pressure is recommended for early diagnosis and management of intra-abdominal hypertension or abdominal compartment syndrome.	R81	R71	x	x
Patients with abdominal compartment syndrome after oeVT of an rAAA should be treated with decompressive laparotomy.	R82	R72	x	x
In the management of open abdomen following decompression for abdominal compartment syndrome after oeVT of rAAA, a vacuum-assisted closure system with mesh-mediated traction and early abdominal closure should be considered.	R83	R73	x	x
For patients undergoing oeVT for rAAAs in whom colonic ischemia is suspected, flexible sigmoidoscopy should be considered to confirm the diagnosis.	R84	x	x	x
Patients with a symptomatic non-rAAA may be considered for a brief period of rapid assessment and optimization followed by urgent repair under optimal conditions (ideally during working hours).	R85	R64	x	x
Symptomatic non-ruptured AAAs should be considered for deferred urgent repair ideally under elective repair conditions	x	R65	x	x
We suggest a door-to-intervention time of <90 min, based on a framework of 30-30-30 min, for the management of the patient with a ruptured aneurysm.	x	x	P41	x
An established protocol for the management of ruptured AAAs is essential for optimal outcomes.	x	x	P41	x
We suggest that patients with ruptured AAAs who require transfer for repair be referred to a facility with an established rupture protocol and suitable endovascular resources.	x	x	P41	x
Immediate repair is recommended in patients with documented aneurysm rupture.	x	x	x	P26
In symptomatic but unruptured AAAs, an optimization of the patient and delayed repair of less than 48 h might be discussed.	x	x	x	P26
An increased abdominal pressure serves as a negative predictive factor for the survival after open repair of a ruptured AAA. Measurement of the intra-abdominal pressure is recommended and, in case of elevated levels (>20 mm Hg), it should immediately be performed in combination with organ dysfunction decompressive surgery. Temporary abdominal closure systems can positively influence outcome.	x	x	x	P27
The set-up of standardized protocols for endovascular treatment of rAAAs including a multidisciplinary approach has been demonstrated successfully and should be employed.	x	x	x	P27
Equipment for EVAR and open repair should be present all the time. A ‘rupture kit’ for the EVAR of rAAAs should be maintained, with an inventory of preferred and most usable stent grafts components with which the treating surgeon has experience. Large-diameter main-body devices with short and long limb lengths should suffice in most emergency cases.	x	x	x	P27
Pre-OP fluid administration should be restricted to a minimum to maintain hypotensive hemostasis.	x	x	x	P28
Patients who are unconscious or in whom a systolic blood pressure cannot be maintained should be immediately transferred to the operating room. The decision to proceed with emergency open repair, placement of an aortic occlusion balloon or invasive imaging studies should depend on the comfort level of the surgeon and conditions of the patient.	x	x	x	P28
In addition to routine physiological monitoring, patients who have undergone EVAR for rAAAs should have hourly bladder pressures recorded to help in the early diagnosis of ACS.	x	x	x	P29

* In the event that a recommendation akin to those previously stated is employed, the table incorporates the most recent formulation. R: Recommendation. P: Page

## References

[1] Wanhainen A., Herzeele I.V., Goncalves F.B., Montoya S.B., Berard X., Boyle J.R., D’Oria M., Prendes C.F., Karkos C.D., Kazimierczak A. (2024). Editor’s Choice—European Society for Vascular Surgery (ESVS) 2024 Clinical Practice Guidelines on the Management of Abdominal Aorto-Iliac Artery Aneurysms. Eur. J. Vasc. Endovasc. Surg..

[2] Moll F.L., Powell J.T., Fraedrich G., Verzini F., Haulon S., Waltham M., Herwaarden J.A.V., Holt P.J., Keulen J.W.V., Rantner B. (2011). Management of abdominal aortic aneurysms clinical practice guidelines of the European society for vascular surgery. Eur. J. Vasc. Endovasc. Surg..

[3] Wanhainen A., Verzini F., Herzeele I.V., Allaire E., Bown M., Cohnert T., Dick F., van Herwaarden J., Karkos C., Koelemay M. (2019). Editor’s Choice – European Society for Vascular Surgery (ESVS) 2019 Clinical Practice Guidelines on the Management of Abdominal Aorto-iliac Artery Aneurysms. Eur. J. Vasc. Endovasc. Surg..

[4] Chaikof E.L., Dalman R.L., Eskandari M.K., Jackson B.M., Lee W.A., Mansour M.A., Mastracci T.M., Mell M., Murad M.H., Nguyen L.L. (2018). The Society for Vascular Surgery practice guidelines on the care of patients with an abdominal aortic aneurysm. J. Vasc. Surg..

[5] Bown M.J., Sweeting M.J., Brown L.C., Powell J.T., Thompson S.G. (2013). Surveillance intervals for small abdominal aortic aneurysms: A meta-analysis. JAMA.

[6] Schmitz-Rixen T., Keese M., Hakimi M., Peters A., Böckler D., Nelson K., Grundmann R.T. (2016). Ruptured abdominal aortic aneurysm—epidemiology, predisposing factors, and biology. Langenbeck’s Arch. Surg..

[7] Harris C.R., Millman K.J., van der Walt S.J., Gommers R., Virtanen P., Cournapeau D., Wieser E., Taylor J., Berg S., Smith N.J. (2020). Array programming with NumPy. Nature.

[8] Hunter J.D. (2007). Matplotlib: A 2D graphics environment. Comput. Sci. Eng..

[9] McKinney W. (2010). Data Structures for Statistical Computing in Python. SciPy.

[10] Virtanen P., Gommers R., Oliphant T.E., Haberland M., Reddy T., Cournapeau D., Burovski E., Peterson P., Weckesser W., Bright J. (2020). SciPy 1.0: Fundamental algorithms for scientific computing in Python. Nat. Methods.

[11] Waskom M. (2021). seaborn: Statistical data visualization. J. Open Source Softw..

[12] Seabold S., Perktold J. (2010). Statsmodels: Econometric and statistical modeling with python. SciPy.

[13] Reimerink J.J., van der Laan M.J., Koelemay M.J., Balm R., Legemate D.A. (2013). Systematic review and meta-analysis of population-based mortality from ruptured abdominal aortic aneurysm. Br. J. Surg..

[14] Sakalihasan N., Michel J.B., Katsargyris A., Kuivaniemi H., Defraigne J.O., Nchimi A., Powell J.T., Yoshimura K., Hultgren R. (2018). Abdominal aortic aneurysms. Nat. Rev. Dis. Prim..

[15] Hoornweg L., Storm-Versloot M., Ubbink D., Koelemay M., Legemate D., Balm R. (2008). Meta Analysis on Mortality of Ruptured Abdominal Aortic Aneurysms. Eur. J. Vasc. Endovasc. Surg..

[16] Badger S., Forster R., Blair P., Ellis P., Kee F., Harkin D. (2017). Endovascular treatment for ruptured abdominal aortic aneurysm. Cochrane Database of Systematic Reviews.

[17] Ulug P., Powell J., Sweeting M., Bown M., Thompson S., Thompson S., Sweeting M., Jones E., Powell J., Ulug P. (2016). Meta-analysis of the current prevalence of screen-detected abdominal aortic aneurysm in women. J. Br. Surg..

[18] Rogers I.S., Massaro J.M., Truong Q.A., Mahabadi A.A., Kriegel M.F., Fox C.S., Thanassoulis G., Isselbacher E.M., Hoffmann U., O’Donnell C.J. (2013). Distribution, determinants, and normal reference values of thoracic and abdominal aortic diameters by computed tomography (from the Framingham Heart Study). Am. J. Cardiol..

[19] Stackelberg O., Björck M., Larsson S., Orsini N., Wolk A. (2014). Sex differences in the association between smoking and abdominal aortic aneurysm. J. Br. Surg..

[20] Jahangir E., Lipworth L., Edwards T.L., Kabagambe E.K., Mumma M.T., Mensah G.A., Fazio S., Blot W.J., Sampson U.K. (2015). Smoking, sex, risk factors and abdominal aortic aneurysms: A prospective study of 18 782 persons aged above 65 years in the Southern Community Cohort Study. J. Epidemiol. Community Health.

[21] Svensjö S., Björck M., Gürtelschmid M., Djavani Gidlund K., Hellberg A., Wanhainen A. (2011). Low prevalence of abdominal aortic aneurysm among 65-year-old Swedish men indicates a change in the epidemiology of the disease. Circulation.

[22] van de Luijtgaarden K.M., Rouwet E.V., Hoeks S.E., Stolker R.J., Verhagen H.J., Majoor-Krakauer D. (2017). Risk of abdominal aortic aneurysm (AAA) among male and female relatives of AAA patients. Vasc. Med..

[23] Wahlgren C.M., Larsson E., Magnusson P.K., Hultgren R., Swedenborg J. (2010). Genetic and environmental contributions to abdominal aortic aneurysm development in a twin population. J. Vasc. Surg..

[24] Joergensen T., Christensen K., Lindholt J., Larsen L., Green A., Houlind K. (2016). High heritability of liability to abdominal aortic aneurysms: A population based twin study. J. Vasc. Surg..

[25] Braet D.J., Taaffe J.P., Singh P., Bath J., Kruse R.L., Vogel T.R. (2021). Readmission and Utilization After Repair of Ruptured Abdominal Aortic Aneurysms in the United States. Vasc. Endovasc. Surg..

[26] D’Oria M., Hanson K.T., Shermerhorn M., Bower T.C., Mendes B.C., Shuja F., Oderich G.S., DeMartino R.R. (2020). Editor’s Choice–Short Term and Long Term Outcomes After Endovascular or Open Repair for Ruptured Infrarenal Abdominal Aortic Aneurysms in the Vascular Quality Initiative. Eur. J. Vasc. Endovasc. Surg..

[27] Malayala S.V., Raza A., Vanaparthy R. (2020). Gender-based differences in abdominal aortic aneurysm rupture: A retrospective study. J. Clin. Med. Res..

[28] McPhee J.T., Hill J.S., Eslami M.H. (2007). The impact of gender on presentation, therapy, and mortality of abdominal aortic aneurysm in the United States, 2001–2004. J. Vasc. Surg..

